# *Euglena gracilis* growth and cell composition under different temperature, light and trophic conditions

**DOI:** 10.1371/journal.pone.0195329

**Published:** 2018-04-12

**Authors:** Yanming Wang, Tuulikki Seppänen-Laakso, Heiko Rischer, Marilyn G. Wiebe

**Affiliations:** VTT Technical Research Centre of Finland Ltd., Espoo, Finland; Stazione Zoologica Anton Dohrn, ITALY

## Abstract

**Background:**

*Euglena gracilis*, a photosynthetic protist, produces protein, unsaturated fatty acids, wax esters, and a unique β-1,3-glucan called paramylon, along with other valuable compounds. The cell composition of *E*. *gracilis* was investigated in this study to understand how light and organic carbon (photo-, mixo- and heterotrophic conditions) affected growth and cell composition (especially lipids). Comparisons were primarily carried out in cultures grown at 23 °C, but the effect of growth at higher temperatures (27 or 30 °C) was also considered.

**Cell growth:**

Specific growth rates were slightly lower when *E*. *gracilis* was grown on glucose in either heterotrophic or mixotrophic conditions than when grown photoautotrophically, although the duration of exponential growth was longer. Temperature determined the rate of exponential growth in all cultures, but not the linear growth rate during light-limited growth in phototrophic conditions. Temperature had less effect on cell composition.

**Cell composition:**

Although *E*. *gracilis* was not expected to store large amounts of paramylon when grown phototrophically, we observed that phototrophic cells could contain up to 50% paramylon. These cells contained up to 33% protein and less than 20% lipophilic compounds, as expected. The biomass contained about 8% fatty acids (measured as fatty acid methyl esters), most of which were unsaturated. The fatty acid content of cells grown in mixotrophic conditions was similar to that observed in phototrophic cells, but was lower in cells grown heterotrophically. Heterotrophic cells contained less unsaturated fatty acids than phototrophic or mixotrophic cells. α-Linolenic acid was present at 5 to 18 mg g^-1^ dry biomass in cells grown in the presence of light, but at < 0.5 mg g^-1^ biomass in cells grown in the dark. Eicosapentaenoic and docosahexaenoic acids were detected at 1 to 5 mg g^-1^ biomass. Light was also important for the production of vitamin E and phytol.

## Introduction

Microalgae, as a source of nutrients, bioactive compounds, biofuels and chemicals, have gained attention from both academia and industry [1–8]. The unicellular, photosynthetic protist *Euglena gracilis*, often considered with eukaryotic algae, contains compounds of commercial interest. *E*. *gracilis* accumulates paramylon (a type of β-1,3-glucan that is unique to euglenoids, particularly *Euglena* species) for energy and carbon storage, especially when grown on organic carbon (i.e. in mixo- or heterotrophic conditions) [4,9]. Similar to other beta glucans, paramylon is considered to have anti-tumor [10] and anti-HIV [11] activity. Paramylon can accumulate to contribute more than 80% of the total cell dry biomass in cells grown in the dark, but accumulated to only 23% of the biomass in phototrophically grown cells [4]. In anaerobic conditions, wax esters become the main storage compounds in *E*. *gracilis* [12]. These esters are composed of medium chain fatty acids and fatty alcohol, which can be converted into biofuels [13,14].

The fatty acid profile of *E*. *gracilis* features a high content (e.g. > 50% of total lipid [15]) of polyunsaturated fatty acids (PUFAs), including the highly desired α-linolenic (ALA), arachidonic (ARA), eicosapentaenoic (EPA) and docosahexaenoic (DHA) acids for human and animal nutrition [15,16]. α-Tocopherol, the most bioactive form of vitamin E is also present in *E*. *gracilis* biomass in a relatively high amount [17]. As a photosynthetic organism, *E*. *gracilis* also contains phytol, a C20 diterpenoid associated with chlorophyll. Phytol has been used to produce synthetic vitamin E, but it and its derivatives have other pharmacological properties of interest to the health industry [18]. Thus, the lipid composition of *E*. *gracilis* makes it suitable for nutritional applications. In addition, the unique cell wall (pellicle) of *E*. *gracilis* is mostly composed of protein [19], making the nutritious *E*. *gracilis* biomass more digestible than other microalgae, which are protected by non-digestible polysaccharides [20]. Around one quarter of the cell biomass may be protein, regardless of whether the cells are grown in the light or dark [12].

*E*. *gracilis* grows in habitats, which vary widely in temperature and pH [3]. It can metabolise many organic carbon sources even in the presence of light [17], and has consequently been studied in autotrophic, and heterotrophic conditions. Photoautotrophic cultivations of *E*. *gracilis* can produce more than 3 g L^-1^ biomass (e.g. [4]). The specific growth rate of *E*. *gracilis* in autotrophic cultures is around 1.1 d^-1^ in favourable growth conditions [21,22]. Ogbonna et al. [23] reported growth rates between 0.9 and 1.1 g L^-1^ d^-1^ during the linear growth phase of *E*. *gracilis* in phototrophic conditions. Higher biomass concentrations and growth rates were achieved in heterotrophic and mixotrophic (photoheterotrophic) conditions [23]. As with other microalgae, in which light is reported to affect the production of carotenoids, polysaccharides and bioactive molecules [24, 25, 26, 27], the presence or absence of light affects cell composition in *E*. *gracilis*, so that mixotrophic growth continues to be of interest in *E*. *gracilis* studies. Some of the cell components are only formed in light, and the formation of some other components (e.g. PUFAs) is enhanced by light [22,28]. Recent studies have shown how the provision of light or organic carbon affect production of lipid-soluble cellular components such as α-tocopherol [4] and unsaturated fatty acids [28].

*E*. *gracilis* is considered to be a promising candidate for development of a photosynthetic biorefinery [3,29] and companies, like Euglena CO. [30], Ltd and Algaeon Ltd, are now producing *E*. *gracilis* biomass and pure β-1,3-glucan for the food and feed industries.

Here we assess growth of *E*. *gracilis* in photo-, mixo- and heterotrophic conditions, and analyse the *E*. *gracilis* cell composition of these cultures at 23 °C as a low temperature, in comparison with 27 or 30 °C as high temperatures. A detailed analysis of the fatty acid composition of *E*. *gracilis* lipids produced in photo-, mixo- and heterotrophic conditions was carried out for cultures grown at 23 °C, as well as for lipids produced at 30 °C in phototrophic conditions. Protein and paramylon content was determined only in phototrophic cultivations (23 and 30 °C), since previous studies have focused on their production in hetero- and mixotrophic conditions [4, 12]. Wax esters were not individually characterised, since all cultures were grown aerobically.

## Material and methods

### Strain, medium, and inocula preparation

Axenic *Euglena gracilis* (NIES-48) was purchased from the National Institute of Environmental Studies of Japan. Cells were maintained in chemically defined medium at room temperature (~22 °C) in non-agitated flasks with low light intensity illumination and sub-cultured every three weeks.

Chemically defined medium was adapted from Ogbonna, Tanaka et al. [17] for macronutrients and Bischoff and Bold [31] for trace elements. For photoautotrophic cultivations, the medium contained (per litre): 0.3 g (NH_4_)_2_SO_4_, 0.08 g (NH_4_)_2_HPO_4_, 0.16 g K_2_HPO_4_, 0.2 g MgSO_4_∙7H_2_O, 0.06 g CaCl_2_∙2H_2_O, 0.5 mg vitamin B1, 0.002 mg vitamin B12, 3.36 mg FeSO_4_∙7H_2_O, 2.88 mg H_3_BO_3_, 4 g Na_2_EDTA∙2H_2_O, 0.314 mg CuSO_4_∙5H_2_O, 1.765 mg ZnSO_4_∙7H_2_O, 0.236 mg MnCl_2_∙2H_2_O, 0.238 mg Na_2_MoO_4_∙2H_2_O and 0.108 mg CoCl_2_∙6H_2_O. For heterotrophic and mixotrophic cultivations, the same medium was modified to provide five-fold more vitamins and trace elements, and 2.5-fold more magnesium to ensure that growth would not be limited by these compounds with the higher biomass concentrations achieved in hetero- and mixotrophic conditions. Specifically, the heterotrophic and mixotrophic medium contained (per litre): 0.3 g (NH_4_)_2_SO_4_, 0.1 g (NH_4_)_2_HPO_4_, 0.2 g K_2_HPO_4_, 0.5 g MgSO_4_∙7H_2_O, 0.06 g CaCl_2_∙2H_2_O, 16.6 ± 0.7 g glucose, 2.5 mg vitamin B1, 0.01 mg vitamin B12, 16.8 mg FeSO_4_∙7H_2_O, 14.4 mg H_3_BO_3_, 20 g Na_2_EDTA∙2H_2_O, 1.57 mg CuSO_4_∙5H_2_O, 8.82 mg ZnSO_4_∙7H_2_O, 1.18 mg MnCl_2_∙2H_2_O, 1.19 mg Na_2_MoO_4_∙2H_2_O and 0.54 mg CoCl_2_∙6H_2_O.

Inocula for bioreactor cultivations were grown in 50 mL photoautotrophic growth medium in 250 mL Erlenmeyer flasks on a shaking platform (Infors AG Switzerland) at 90 rpm, 24 °C. One or two fluorescent tubes (8 W) were placed on the edge of the shaker to provide light intensities of 100 to 150 μmol photons m^-2^s^-1^ on culture surfaces. Light intensity was measured with a quantum sensor connected to a radiometer (Li-Cor. Inc. USA). Inocula were cultivated until the optical density at 780 nm (OD_780_) was 0.3.

### Stirred tank (photobioreactor) cultivations

Sartorius BioStat B 2.5 L glass vessel bioreactors, with a water jacket, (Sartorius AG, Germany) were set up as described by Wang et al. [32]. Two-litre working volume cultures were maintained at pH 6.0 (with 1 M H_3_PO_4_ or 1 M NaOH for titration), 200 rpm agitation, and 0.1 volume of air per volume culture per minute (vvm) aeration with CO_2_ enriched air (1.8% CO_2_, 20.9% O_2_ and 77.7% N_2_). Cultures were maintained at pH 6.0 to prevent precipitation of salts in the medium, since this was within the optimal pH range (between pH 3 and 6) for *E*. *gracilis* [21]. Cultivations were carried out at 23 °C (low temperature), 27 °C or 30 °C (high temperatures, Table 1). The light intensity of the cultures was adjusted by using either two or three externally mounted fluorescent lamps (11 W, 20 cm, with reflective back cover, Lival Oy, Finland), vertically mounted at equal distances around the reactor vessel, each providing light intensities of approx. 400 μE m^-2^ s^-1^ at the inner surface of the reactor directly facing the lamps. Light intensity is thus described as either 2 or 3 x 400 μE m^-2^ s^-1^, to reflect the illumination provided to the surface of the cultures. The same bioreactors were wrapped in foil to eliminate stray light for heterotrophic cultures (at 23 and 27 °C).

**Table 1:** Cultivation conditions and analyses carried out from each cultivation.

	Cultivation	1	2	3	4	5	6	7	8
**Conditions**	**Temperature (°C)**	**23**	**23**	**23**	**23**	**27**	**27**	**30**	**30**
	**Light (x 400 μE m**^**-2**^ **s**^**-1**^**)**	**2**	**2**	**0**	**3**	**2**	**0**	**3**	**3**
	**Glucose added**	**-**	**+**	**+**	**-**	**+**	**+**	**-**	**-**
**Analyses**	**Specific growth rate**	Fig 1	Fig 2	Fig 2	Fig 1	Fig 2	Fig 2	Fig 1	Fig 1
	**Linear growth rate**	Fig 1	Fig 2	Fig 2	Fig 1	Fig 2	Fig 2	Fig 1	Fig 1
	**Lipids (total)**	**+**	**-**	**-**	**+**	**-**	**-**	**+**	**-**
	**Lipids (composition)**	**-**	**+**	**+**	**+**	**-**	**-**	**(+)**	**+**
	**Protein and paramylon**	**-**	**-**	**-**	**+**	**-**	**-**	**(+)**	**+**
	**Figure legend**	**○**	**⊕**	**●**	**□**	**◇**	**◆**	**Δ**	**∇**

+ indicates that glucose was added to the cultivation or that the specified analysis was carried out. (+) indicates the analysis was carried out only at the end of the cultivation.—indicates that glucose was not added (photoautotrophic culture) or that the indicated analysis was not carried out. The figure in which data on specific and linear growth rate determination is indicated.

Cultivations 7 and 8 provided independent replicates, although not all analyses were carried out for each replicate.

The composition of the CO_2_ enriched gas stream and the exhaust gas (percent O_2_, CO_2_ and N_2_) from the cultivations was analysed by a photoacoustic IR gas analyser (Innova-1313/LumaSense, United States), with air as reference or a Prima Pro Process mass spectrometer (Thermo Scientific, Winsford, UK) calibrated with 3% CO_2_ in Ar, 5% CO_2_ with 0.99% Ar and 15% O_2_ in N_2_, 20% O_2_ plus 20% Ar in N_2_, and 0.04% ethanol in N_2_. The CO_2_ enriched gas stream composition was measured before inoculating each cultivation.

### Analyses

#### Cell biomass

Cell biomass was measured as OD_780_ and dry weight. OD_780_ was used to monitor increase in biomass at low cell densities and was measured in triplicate against deionised water using an infrared spectrophotometer (Hitachi U-2000, Japan). Since phototrophic algal cultures only grew exponentially at low biomass concentrations, OD_780_ was used to calculate specific growth rates in these cultures. For dry weight determination, at least 3 mL of culture was centrifuged (3000 rpm 10 min), and the pellet was washed twice with deionised water. During the washing step, the cell biomass was transferred to a 2 mL pre-dried (105 °C) and weighed microcentrifuge tube. The tube containing the cell biomass was dried by lyophilisation (Christ^®^ lyophiliser, Sartorius AG, Germany) and re-weighed. Dry weight was determined for a minimum of 2 replicates (for low biomass concentrations which required large sample volumes), but usually for 4 and occasionally 6 replicates, depending on the volume required to obtain an accurate measurement. The dry algal biomass was used for lipid, protein and paramylon content analyses. Supernatant was retained for ammonium and glucose analysis.

#### Ammonium analysis

Ammonium was measured using an ion selective ammonium electrode (C-CIT AG, Switzerland) with a detection limit of 0.3 mmol L^-1^.

#### Total lipid extraction and analysis

Total lipid content was determined gravimetrically after chloroform-methanol (2:1) extraction adapted from Folch *et al*. [33], as described by Wang *et al*. [32].

#### Fatty acid analysis by gas chromatography

Freeze-dried samples (2–25 mg) were homogenised with a Retsch Mixer Mill homogenizer (Retsch GmbH, Haan, Germany) for 2 x 30 s (20 Hz, 2 steel grinding balls ø 3 mm) and extracted twice with 1 ml chloroform:methanol (2:1). The chloroform-methanol extracts, containing the lipids, were combined to obtain the total lipids and 30.45 μg C17:0 triacylglycerol (TG, 17:0/17:0/17:0) and 15.0 μg C17:0 free fatty acid (FFA) were added to a 300 μl aliquot before removal of the solvent under nitrogen flow. Lipids were re-dissolved in 1 ml petroleum ether (bp 40–60 °C). Transesterification was performed by adding 500 μl of 0.5 N sodium methoxide in methanol and two boiling stones to the solubilised lipids, then boiling at 45 °C for 5 min. Fifteen percent (w/v) NaHSO_4_ (1 ml) was added to acidify the samples, after which methyl esters and free fatty acids were extracted with petroleum ether. The separated petroleum ether layer was evaporated and the residue re-dissolved in 150 μl hexane. Fatty acid methyl esters were analysed on an Agilent 7890A GC equipped with an Agilent FFAP silica capillary column (25 m × 0.2 mm × 0.3 μm). Hydrogen was used as carrier gas with a split ratio of 20:1. The oven temperature was increased from 70 °C (2 min) to 235 °C at a rate of 10 °C/min, with a total run time of 30 min.

After quantifying fatty acid methyl esters (FAME) by GC, the same samples were trimethylsilylated to determine FFAs and other polar compounds by GC-MS (Agilent 7890A GC combined with 5975C MSD). The solvents were evaporated and samples re-dissolved in 50 μl dichloromethane and silylated with 25 μl *N*-Methyl-*N*-(trimethylsilyl)trifluoroacetamide (MSTFA) for 20 min at 80 °C. The samples were analysed on an Agilent DB-5 silica capillary column (30 m x 0.25 mm x 0.25 μm). The split ratio was 20:1 and the oven temperature programme from 70 °C (1 min) to 240 °C at a rate of 10 °C/min, the total run time was 30 min. The samples (2 μl) were injected by a Gerstel MPS injection system and the data were collected in EI mode (70 eV) at a mass range of m/z 50–600.

#### Extraction and measurement of protein and paramylon

Protein extraction and quantification was based on the methods developed by Slocombe *et al*. [34]. Approximately 5 mg freeze-dried *E*. *gracilis* biomass was weighed and re-suspended in 250 μl 10% (w/v) trichloroacetic acid (TCA). The suspension was ultrasonicated for 15 min (Fritsch ultrasonic cleaner, FRITSCH GmbH, Germany) and then incubated in a 65 °C water bath for 30 min. The samples were vortexed several times during the incubation to ensure complete cell disruption. After cooling to room temperature, samples were centrifuged at 15,000 g, 4 °C, for 20 min. The pellets were resuspended in 0.5 ml alkaline solution containing 20 g L^-1^ Na_2_CO_3_ and 4 g L^-1^ NaOH to dissolve the proteins. The suspension was centrifuged to remove the non-soluble particles, including paramylon, from the protein extract. Protein was quantified using the DC protein assay kit (Bio-RAD), based on the Lowry method [35].

*P*aramylon extraction was adapted from Barsanti et al. [9]. Protein free pellets from the extracted *E*. *gracilis* cells were washed with 3 ml ethanol, resuspended in 3 ml sodium dodecyl sulfate (SDS) solution (2 g L^-1^) and incubated at 37 °C for 24 h. Solids were collected by centrifugation and the supernatant discarded. SDS washing was repeated without incubation. The paramylon pellets were washed twice with 3 ml distilled water, then dissolved in 2 ml NaOH (1 M). The concentration of paramylon was determined using the phenol-sulfuric acid method described by Albalasmeh et al. [36].

## Results

### Autotrophic growth of *E*. *gracilis*

**Fig 1:**
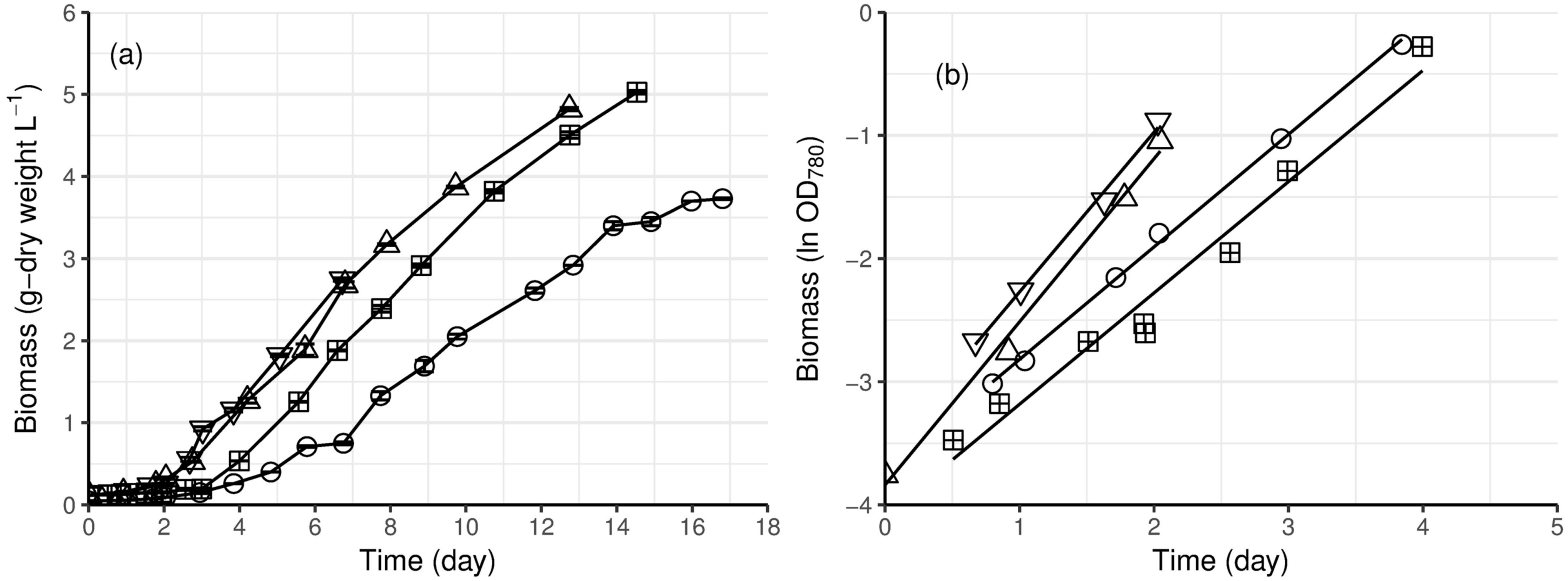
Growth of photoautotrophic cultures of *E*. *gracilis* at 23 (circle) or 30 °C (upward and downward triangles) with two (square) or three (circle and triangles) light sources of 400 μE m^-2^ s^-1^ intensity (2 x 400 or 3 x 400 μE m^-2^ s^-1^). (a), Growth curves measured by cell dry weight. Error bars (which were generally smaller than the symbol) represent the standard error of the mean (n = 2–6). (b), Natural logarithms (ln) of cell density measured by OD_780_, and the least square regression lines.

*E*. *gracilis* initially grew exponentially in photoautotrophic batch cultures (Fig 1b), with no apparent lag phase. The exponential phase lasted for approximately 2 to 4 days (depending on the temperature), until there was about 0.5 g l^-1^ biomass, after which the cultures became light limited and increase in biomass was linear (Fig 1a). The cultures were stopped before stationary phase. At 23 °C, the specific growth rate of *E*. *gracilis* during the exponential phase was 0.90 d^-1^, regardless of the light intensity. At 30 °C with 3 x 400 μE m^-2^ s^-1^ light it was 40% higher (1.30 d^-1^, Table 1 and Fig 1b) than at 23 °C.

The growth rate of *E*. *gracilis* in the linear phase (0.50 g L^-1^ d^-1^, Table 2, Fig 1a), i.e. after the 2–4 days exponential growth, was similar at 23 or 30 °C, for cultures at 3 x 400 μE m^-2^ s^-1^ light.

**Table 2:** Specific growth rates (μ) and volumetric growth rates (r-lin) of photoautotrophic cultures of *E*. *gracilis* at 23 and 30 °C with 2 x 400 or 3 x 400 μmol m^-2^ s^-1^ light.

Light (μmol m^-2^ s^-1^)	2 x 400	3 x 400	3 x 400	3 x 400
T (°C)	23	23	30	30
μ (d^-1^)	0.91±0.05	0.90±0.16	1.31±0.35	1.30±0.28
r-lin (g L^-1^d^-1^)	0.35±0.04	0.50±0.04	0.49±0.06	0.52±0.09

The data are presented with ± 95% confidence interval

Comparison of the linear growth rates with more (3 x 400 μE m^-2^ s^-1^) or less (2 x 400 μE m^-2^ s^-1^) light was made at 23 °C and demonstrated that the linear growth rate (0.35 g L^-1^d^-1^) was lower with less light provided than with 3 x 400 μE m^-2^ s^-1^ (Table 2). Providing more light also allowed more biomass to be produced: after 13 or 14 days, 5.0 g L^-1^ biomass had been produced in cultures receiving 3 x 400 μE m^-2^ s^-1^ light; while the culture with 2 x 400 μE m^-2^ s^-1^ light generated 3.7 g L^-1^ biomass in 17 days.

### Heterotrophic and mixotrophic growth of *E*. *gracilis*

**Fig 2:**
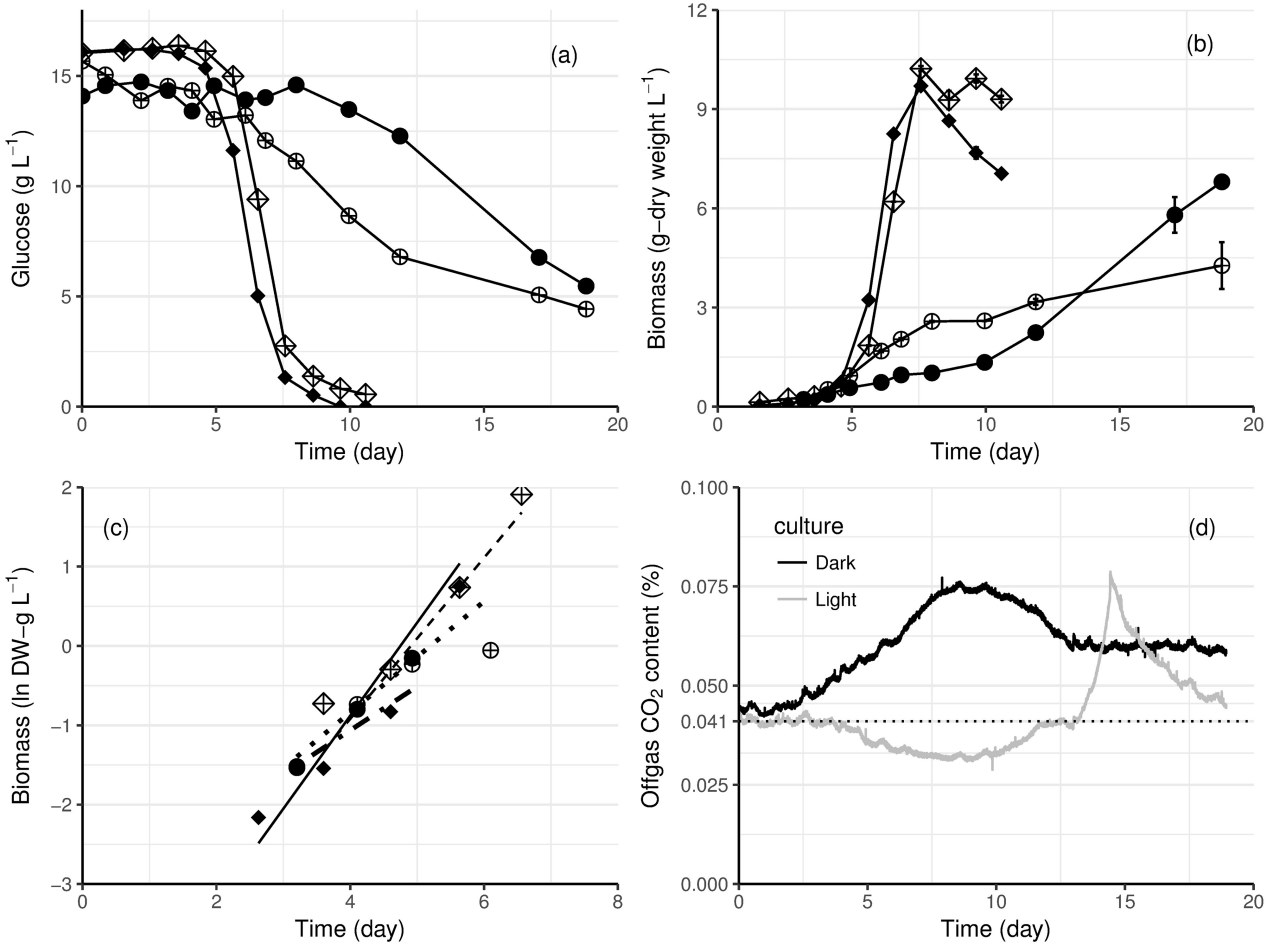
Growth and glucose consumption of *E*. *gracilis* in heterotrophic and mixotrophic conditions. (a) Glucose consumption and (b) biomass production of heterotrophic (solid symbols) and mixotrophic (symbols with cross) cultures of *E*. *gracilis*. Cultures were grown at 27 °C (diamonds) and 23 °C (circles). Error bars represent ± standard error of the mean (n = 4 for cultures at 23 °C, n = 2 for cultures at 27 °C). (c) The exponential growth phase is shown by plotting the biomass dry weight on a logarithmic scale. The least square regression lines are shown by the dashed lines. (d), Carbon dioxide content in the off gas in heterotrophic (black) and mixotrophic (grey) cultures of *E*. *gracilis* at 23 °C. The dotted line indicates the CO_2_ content of air, as measured with the MS.

*E*. *gracilis* cells in both heterotrophic and mixotrophic conditions only started to consume glucose after an adaptation phase (up to 8 days for the mixotrophic culture at 23 °C, Fig 2a), reflecting that the inocula had been grown photoautotrophically in medium without glucose. Glucose consumption was observed 1–2 days after biomass began to increase in the mixotrophic cultures (Fig 2). No net CO_2_ production occurred during initial growth in light, whereas CO_2_ production was correlated to growth throughout the cultivation in the dark (cf. Fig 2b and 2d). The specific growth rate of heterotrophic cultures was higher than that of the mixotrophic cultures (Table 3). However, the final cell densities of mixotrophic cultures at both temperatures were greater than in heterotrophic cultures (Fig 2b).

**Table 3:** Specific growth rates (μ) during exponential phase of mixotrophic (primarily phototrophic growth) and heterotrophic cultures of *E*. *gracilis* at 23 and 27 °C.

μ (d^-1^)	Mixotrophic	Heterotrophic
23 °C	0.55±0.14	0.70±0.34
27 °C	1.02±0.41	1.17±0.36

The data are presented with ± 95% confident interval.

The specific growth rate of *E*. *gracilis* was higher at 27 °C than at 23°C, in both heterotrophic and mixotrophic conditions (Table 3 and Fig 2c). The higher temperature also shortened the adaptation phase for mixotrophic culture to consume glucose (Fig 2a).

### *E*. *gracilis* lipid content and profile

**Fig 3:**
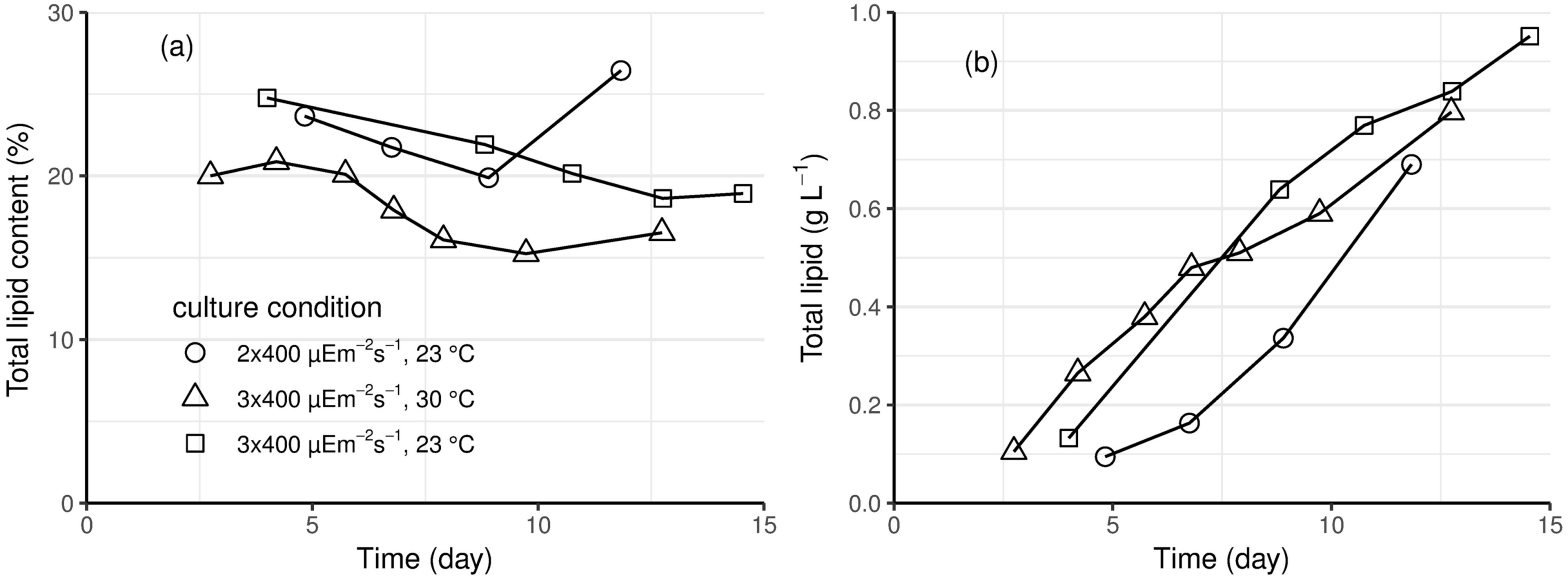
Total lipid (a) content, as percentage of dry weight, and (b) concentration of *E*. *gracilis* biomass generated in autotrophic conditions. Cultures grown at 23 °C (circle and square) with 2 x 400 (circle) or 3 x 400 μmol m^-2^ s^-1^ (square) light or at 30 °C (triangle) with 3 x 400 μmol m^-2^ s^-1^ light.

*E*. *gracilis* total lipid content was relatively constant during both exponential and linear growth phases in photoautotrophic cultures (Fig 3a). At 23 °C, the *E*. *gracilis* cells contained more lipids (on average 22%) than at 30 °C (on average 18%). However, since the total lipid produced in a culture is a function of the lipid content of the biomass and the biomass concentration, the total concentration of lipids of the photoautotrophic cultures receiving the same light intensity (3 x 400 μE m^-2^s^-1^) were similar, but less lipid was produced when less light was provided (Fig 3b).

**Fig 4:**
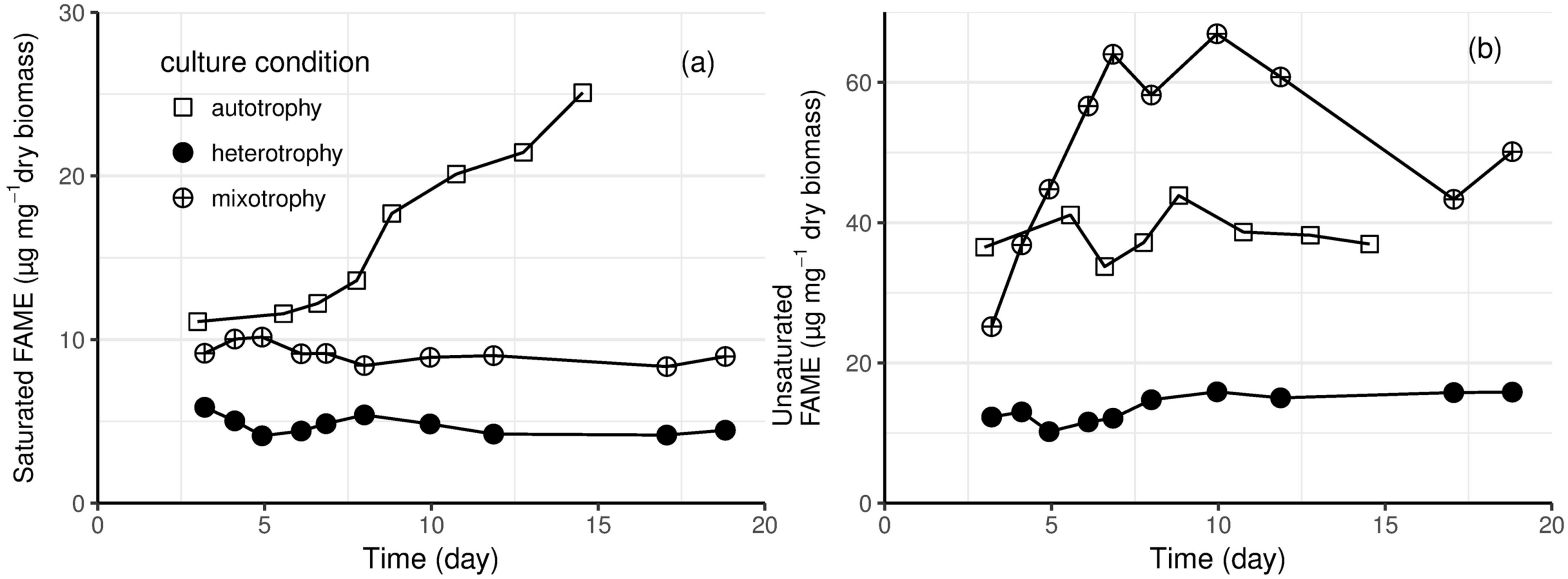
The (a) saturated and (b) unsaturated FAME content of *E*. *gracilis* grown in photoautotrophic (square), heterotrophic (solid circle) and mixotrophic (circle with cross) batch cultivations at 23 °C.

Total lipids measured gravimetrically in photoautotrophic cultures contained only 30% fatty acids that could be transesterified to fatty acid methyl esters (FAMEs) and less than 5% free fatty acids; the remaining ca. 65% probably contained lipophilic compounds such as wax esters, pigments (including chlorophyll and carotenoids), phytol, and unidentified compounds. Fig 4 shows the saturated and unsaturated FAME content of *E*. *gracilis* biomass in photoautotrophic, heterotrophic and mixotrophic batch cultivations at 23 °C. In all conditions, *E*. *gracilis* biomass contained more unsaturated than saturated fatty acids. *E*. *gracilis* biomass from heterotrophic cultures contained the lowest amount of fatty acids (both saturated and unsaturated), and their content remained constant throughout the cultivation. The maximum unsaturated fatty acid content (67 μg mg^-1^) was obtained in mixotrophic culture at the end of the exponential phase (ca. 7 days after inoculation; Fig 4b). The photoautotrophic culture (23 °C) accumulated 25 μg mg^-1^ saturated fatty acids by the end of the cultivation, which was higher than in mixotrophic and heterotrophic cultures (Fig 4a).

**Fig 5:**
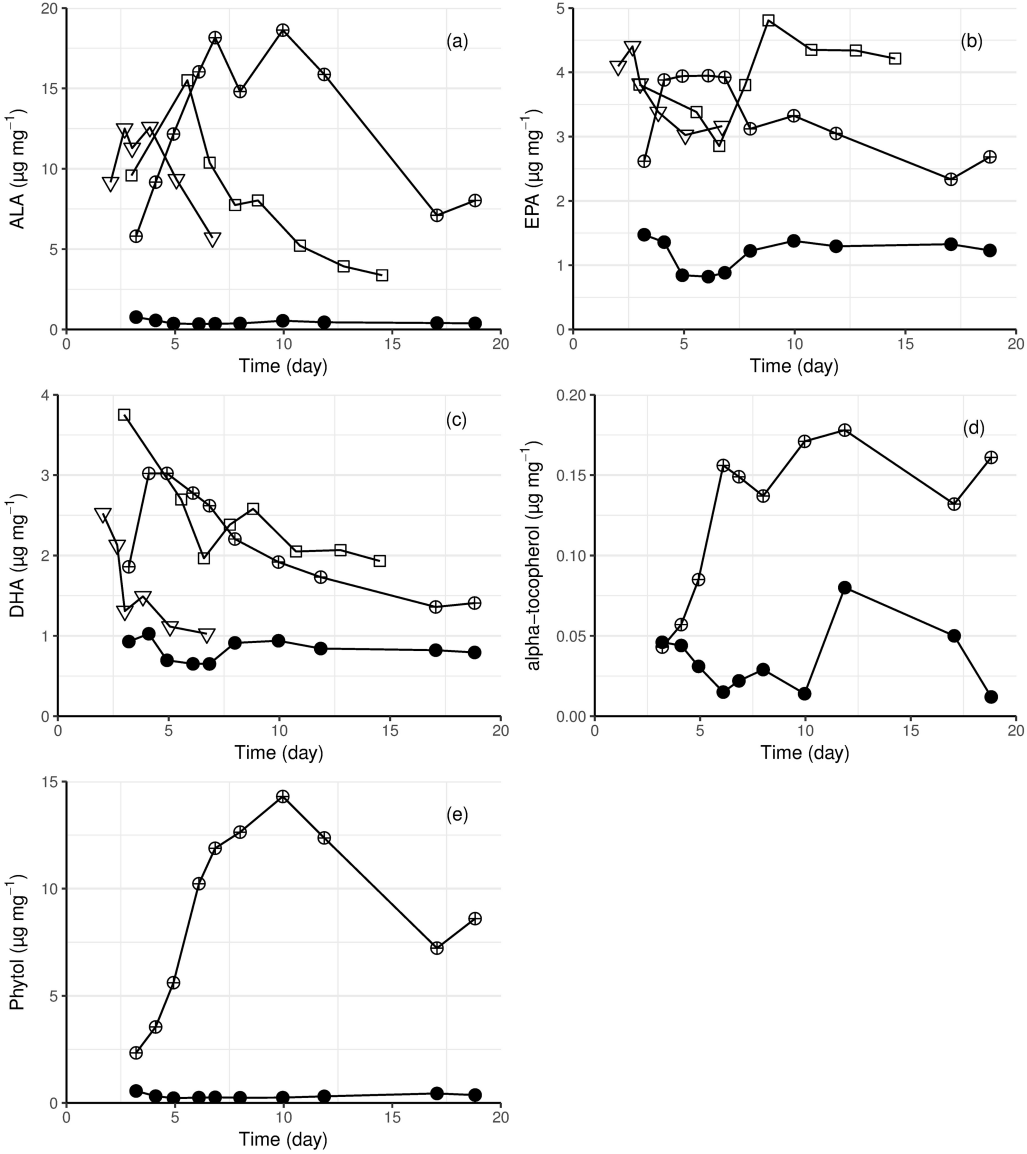
The content of (a) ALA, (b) EPA, (c) DHA, (d) α-tocopherol and (e) phytol in *E*. *gracilis* biomass from autotrophic (23 °C square, 30 °C triangle), heterotrophic (23 °C solid circle) and mixotrophic (23 °C circle with cross) cultivations.

In light grown *E*. *gracilis*, the most abundant PUFA was ALA (see supporting information files S1 and S2 Figs for comparison with other fatty acids). Its maximum concentration (18 mg g^-1^) was obtained from the mixotrophic culture at 23 °C. ALA content was low (< 0.5 mg g^-1^) throughout the heterotrophic culture (Fig 5a). Long chain PUFAs, such as EPA and DHA, were present in *E*. *gracilis* biomass from all cultures. The maximum concentrations of EPA and DHA were 4.8 and 3.8 mg g^-1^, respectively, in cells from the photoautotrophic culture (Fig 5b and 5c). The EPA content was similar among light grown cultures (autotrophic and mixotrophic), but it was one third lower in the heterotrophic culture (Fig 5b). The heterotrophically grown biomass also contained the lowest amount of DHA. Both light grown cultures at 23 °C contained more DHA than the culture at 30 °C (autotrophic). In all light grown cultures the DHA content decreased during cultivation (Fig 5c).

Wax esters were detected in both mixotrophically (maximum 2.6 mg g^-1^ cell dry weight) and heterotrophically (maximum 3.2 mg g^-1^ cell dry weight) grown cells. The majority were esters of C14 and C16 fatty acids and fatty alcohols (14:0–14:0, 14:0–16:0 and 16:0–16:0). In mixotrophic cultures the α-tocopherol content increased during exponential growth (from 0.04 to 0.18 mg g^-1^), whereas in the heterotrophic culture, it decreased to 0.01 mg g dry biomass^-1^ (Fig 5d). Phytol, associated with photosynthesis, was present in concentrations about 80-fold higher than that of α-tocopherol in mixotrophically grown *E*. *gracilis* cells (up to 14 mg g^-1^ biomass), but not in the heterotrophically grown ones (< 0.5 mg g^-1^ biomass). The phytol content was highest (1.4% of the cell dry weight) at the end of the exponential growth phase. Its content gradually decreased during the linear growth phase (Fig 5e). The content of other fatty acids, measured as FAME and ranging in length from C12:0 to C22:6, in the cells is shown in supporting information S1 and S2 Figs.

### Paramylon and protein in photoautotrophically grown *E*. *gracilis*

**Fig 6:**
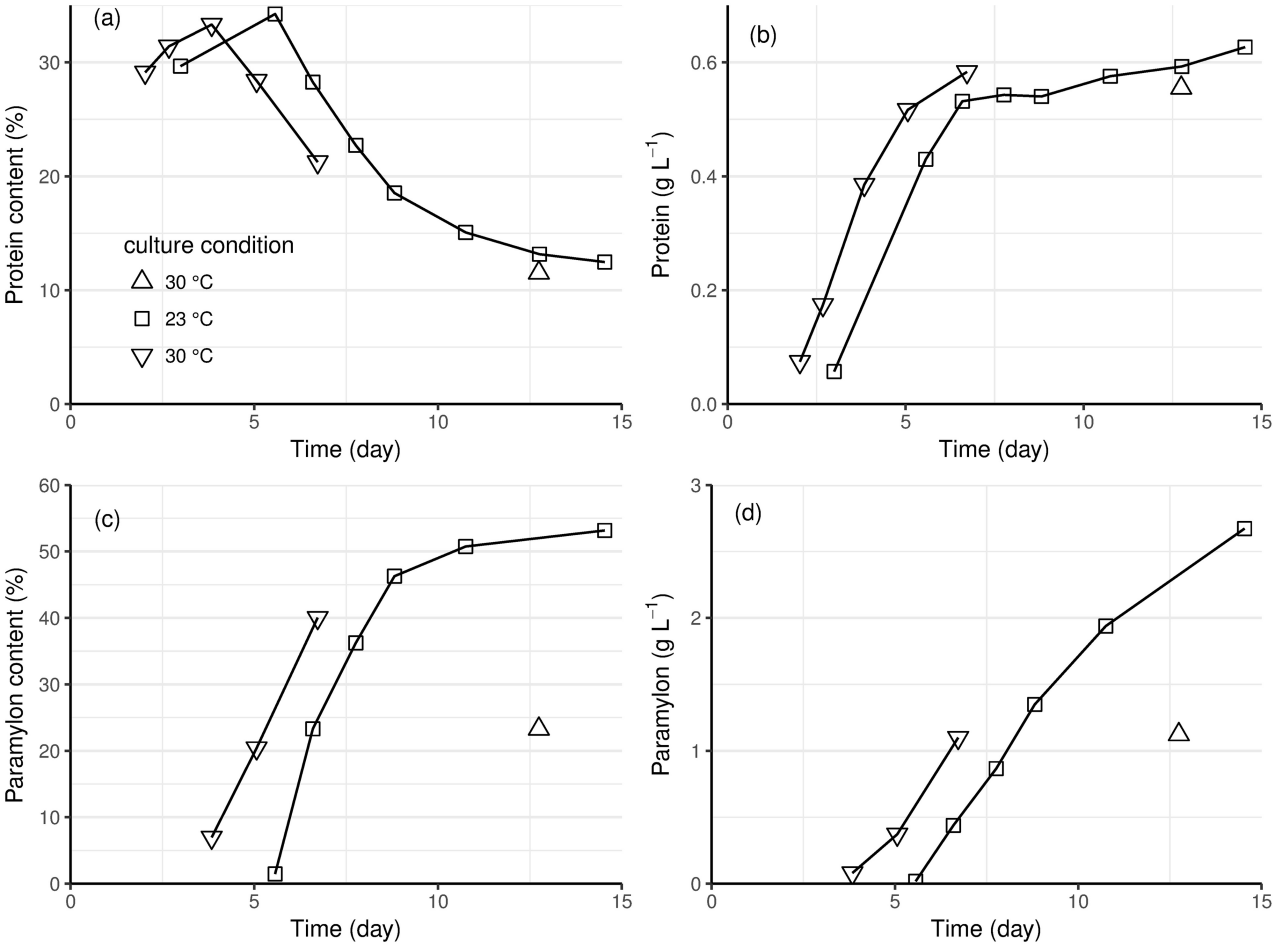
The content (as percent, a and c) and concentration (b and d) of protein (a and b) and paramylon (c and d) in photoautotrophically grown *E*. *gracilis*. Cultures grown at 23 °C (square) and at 30 °C (up and down triangles) with 3 x 400 μmol m^-2^ s^-1^ light. One culture at 30 °C provided samples during the initial 6 days of growth, whereas the second culture included only an end-point sample, the data from which is included here for comparison with the longer cultivation at 23 °C.

*E*. *gracilis* cells grown in autotrophic conditions contained 12–34% protein. The cell protein content (as a proportion of cell dry weight) was highest during the exponential growth phase (observed maximum protein content was 34.2% at day-6 of the culture at 23 °C, Fig 6a). Both protein content and cell biomass increased during the exponential phase, so total protein concentration in the culture increased rapidly (Fig 6b). The total protein concentration of the culture remained constant during the linear growth phase, as the relative proportion of protein in the cells decreased, and the biomass concentration increased. Protein content was similar in the cultures at 23 and 30 °C (Fig 6).

The paramylon content of the cells was 1.5% (23 °C) or 7.0% (30 °C) at the end of the exponential growth phase, and increased during the linear growth phase. After 9 days, 46.3% of the total cell dry biomass was paramylon, increasing to 53.2% at the end of the cultivation in the culture at 23 °C (Fig 6c). The total amount of paramylon in the cultures increased throughout the cultivation, as also observed at 30 °C (Fig 6d).

## Discussion

The specific growth rate of photoautotrophic *E*. *gracilis* culture was 45% higher at 30 °C than at 23 °C (Table 2), as observed in previous studies [22,37]. The optimal growth temperature for *E*. *gracilis* is between 27 and 30 °C [22]. However, we observed that temperature only affected the specific growth rate (i.e. the exponential growth phase), in which all substrates, including light, were sufficient, and not the light-limited volumetric growth rate (Table 2). For photoautotrophic microalgal cultures, sustaining exponential growth is dependent on sufficient light penetration, and is thus limited by cell density and reactor geometry. In the stirred tank photobioreactor used here, provided with 3 x 400 μE m^-2^s^-1^ light, exponential growth of *E*. *gracilis* stopped when the biomass reached 0.5 g L^-1^ (Fig 1). Lower overall light intensity did not affect the specific growth rate during the exponential phase, but the critical biomass level was lower (< 0.3 g L^-1^) when less light (2 x 400 μE m^-2^s^-1^) was provided than with 3 x 400 μE m^-2^ s^-1^ (Fig 1a, Table 2). A similar critical biomass level was observed by Li et al. in *Scenedesmus sp*. cultures [38]. Above the critical biomass concentration, the culture is light limited because of self-shading, but linear increase in biomass occurs [39]. In the linear growth phase, the specific growth rate gradually decreases, as the self-shading increases, and the volumetric rate of biomass production becomes the parameter by which growth is evaluated. That the volumetric biomass production rate was affected by light provision, but not temperature (Table 2), confirmed that light availability was the limiting factor for *E*. *gracilis* growth during this phase. Various reactor designs for improving light transfer in photobioreactors have been tested, which include internal illumination, enhanced mixing and shortening the light path [40], but a culture inevitably reaches a critical biomass concentration, at which light limitation results in linear growth. Thus the temperature of operation (within the range permissive of growth) is unlikely to be critical for *E*. *gracilis* biomass production at commercial scales, which would be light limited most of the time. This would allow various geographical regions with both warm and cool climates to be used for *E*. *gracilis* cultivation [30].

When grown heterotrophically and mixotrophically, exponential growth of *E*. *gracilis* was no longer limited by light. As in photoautotrophic cultures, the specific growth rate was affected by temperature, but temperature also affected the time required for adaptation to glucose utilization in mixotrophic cultures. Low temperature (23 °C) delayed growth on glucose (for 8 days). Light is also known to inhibit glucose transport in *E*. *gracilis* [28,41], although the mechanism of the inhibition remains unclear and the inhibition only applies to glucose, not other organic carbon sources. In *E*. *gracilis*, photoautotrophic and heterotrophic metabolic activities are reported to occur simultaneously only at very low light intensity [23]. Nicolas et al. observed no glucose consumption for 6 to 7 days when *E*. *gracilis* was grown in nitrogen-limited conditions with only 600 lux (~8 μmol m^2^ s^-1^) light and longer delays in its consumption in the presence of higher light intensity [41]. In the current study, we provided 3 x 400 μmol m^2^ s^-1^ and observed a delay of 8 days before glucose consumption started at 23 °C, reduced to only 4 days at 27 °C (Fig 2). Although the specific growth rate in mixotrophic cultures was slightly lower than in heterotrophic cultures (Table 3), the final biomass concentration was higher in the mixotrophic conditions, as observed by Yamane et al. [42] and Zeng et al. [43]. Providing light to the mixotrophic culture at 27 °C was sufficient to prevent cell death and lysis after glucose had been consumed.

Despite the inhibition of glucose utilisation by light, *E*. *gracilis* has been grown in mixotrophic [44] as well as heterotrophic (e.g. to produce 39.6 g L^-1^ biomass [17]) conditions to generate high biomass concentrations. Although providing light incurs cost, mixotrophy remains of interest since specific products, such as unsaturated fatty acids, are only produced or are produced in larger amounts when cultures are exposed to light [42,43,45].

In general, *E*. *gracilis* accumulates much less lipid than oleogenic microalgal species like *Chlorella* (30–57% lipid) [32] and *Nannochloropsis* (26–42% lipid) [46], but is known to contain a large proportion of unsaturated lipids [47]. Although lipid content as high as 37% has been reported from photoautotrophic conditions [48], the lipid content is typically only 8 to 18% of the cell biomass [43,44]. Neutral lipids are not the main storage compounds of *E*. *gracilis*, so much of the fatty acids extracted from *E*. *gracilis* are derived from the phospholipids of the cell and organelle membranes [44,49]. Only 11–18% of the lipids in *E*. *gracilis* are triacylglycerols [49]. Considerable amounts of lipophilic compounds other than fatty acids are included in gravimetric measurements of lipid content [48,50]. In the present study, about 20% of the dry biomass of cells grown in photoautotrophic conditions was extracted as total lipid (gravimetric measurement), less than half of which was detected as fatty acids.

Most of the unsaturated fatty acids In *E*. *gracilis* are found in chloroplast membranes [48] and the unsaturated fatty acid content of the cells is affected by the activity and amount of chloroplasts, which, in turn, are dependent on light availability. Up to 80% of the lipids in light grown cells may be unsaturated, while the proportion in dark grown cells was 32% [43,51]. In the current study, we observed 78 to 88% unsaturated fatty acids in cells grown in light and around 72% in cells grown without light. The content of both saturated and unsaturated fatty acids was essentially constant in the dark grown cells, but the content of unsaturated fatty acids initially increased in mixotrophic conditions (Fig 4). A subsequent decrease in unsaturated fatty acids content corresponded to the start of glucose utilization. The mixotrophically grown *E*. *gracilis* contained up to five times more unsaturated fatty acids than the cells grown in the dark (Fig 4b). The same trend has also been observed by Schwarzhans et al. [28] and Zeng et al. [43].

ALA was the major PUFA in light grown *E*. *gracilis*; it was nearly absent in dark grown cells (Fig 5), as previously observed [28,43,48]. Two long-chain omega-3 fatty acids important for human physiology, EPA and DHA, were also present in higher amounts in light grown cultures than in heterotrophic cultures (Fig 5); i.e. the synthesis of omega-3 fatty acids, like other unsaturated fatty acids in *E*. *gracilis*, was stimulated by light. Growth at 23 °C, rather than 27 °C also had a positive effect on DHA production. Although *E*. *gracilis* did not show higher productivity of any single PUFA in the conditions tested here, compared to other PUFA producing microalgal species, *E*. *gracilis* contains all basic omega-3 and omega-6 fatty acids, making its biomass suitable for high quality feed [52].

In the current study, α-tocopherol content was also light dependent, but the concentration was lower than observed by Grimm et al. in either heterotrophic or mixotrophic conditions [4] and more comparable to that observed by Kusmic et al. [53]. α-Tocopherol production is associated with photosynthetic organisms [54], but can be produced in mitochondria as well as in chloroplasts [53]. None-the-less, production of α-tocopherol, even in *E*. *gracilis* lacking chloroplasts is stimulated by light [53].

Although *E*. *gracilis* is expected to synthesise wax esters in anaerobic conditions by converting storage paramylon to wax esters [55], we observed that wax esters were constantly present at low concentrations in aerobic heterotrophic and mixotrophic cultures of *E*. *gracilis*.

Kunne and de Groot found that protein synthesis in *E*. *gracilis* was dependent on both temperature and light, with high light and low-temperature favouring protein synthesis [56]. However, we observed similar protein production at 30 and 23 °C, with the protein content being dependent on the stage of the culture, not the temperature. Protein content was highest at the end of the exponential phase, i.e. before light limitation occurred. During the light-limited, linear growth phase, *E*. *gracilis* protein content decreased (Fig 6). The total protein content of the population increased very slowly during this phase, suggesting that little new protein was synthesized. Carell et al. also observed that cells in older cultures contained about half the maximum protein content per cell [57].

Unlike protein, most paramylon accumulated during the linear growth phase in photoautotrophic *E*. *gracilis* cultures (Fig 6). Up to ~50% of the cell biomass, 2.7 g L^-1^, was paramylon at 23 °C, which was comparable to that reported for heterotrophic paramylon production (35 to 85% of cell biomass) [28,12]. This was much higher than previously reported for a photosynthetic culture of *E*. *gracilis* (23% of cell biomass) [4]. Accumulation of paramylon in heterotrophic and mixotrophic cultures has been associated with exponential growth [9], whereas we observed accumulation of paramylon in phototrophic conditions only after the cells and shifted into a slower, linear increase in biomass (*cf*. Figs 1 and 5). It may be that Grimm et al. [4] did not observe high accumulation of paramylon in their photoautotrophic cultures because they stopped sampling too early or because of limitations of growth in the linear phase in shaken flasks, compared to growth in a photobioreactor. As with protein synthesis, paramylon accumulation was not dependent on temperature, but rather on the stage of the culture. Our result indicated that *E*. *gracilis* cells can be harvested to provide either protein or paramylon, but that the cultivation process should be optimised for the specific target product, to maximise the yield and productivity.

## Conclusions

*E*. *gracilis* grew well in photoautotrophic, mixotrophic and heterotrophic conditions at 23 °C, with mixo- and heterotrophic conditions primarily providing a benefit in producing high biomass concentrations. Providing light, whether in photoautotrophic or mixotrophic cultures, however, strongly influenced the cell composition. Total unsaturated fatty acids, omega-3 fatty acids, and α-tocopherol content in the cells was higher in the presence of light than in its absence. However, saturated fatty acids were still highest in cells grown in phototrophic conditions.

Increasing the temperature provided only limited benefit to phototrophic cultures, since the temperature only affected the relatively short exponential growth phase. In phototrophic cultures, temperature also had little impact on the production of protein, paramylon or total lipid, although the production of some specific fatty acids, such as DHA were lower at high than at low temperature. In contrast, cultivation temperature was important when the cells grew on organic carbon, since the exponential phase was sustained until the organic carbon was consumed.

The above-mentioned *E*. *gracilis* cell components are all either essential nutrients or compounds that promote human and animal health. Thanks to their ability to produce this unique range of compounds, dried *E*. *gracilis* whole cells and *E*. *gracilis* extracts are already on the market (sold by companies like Euglena Ltd [30]. Algaeon Inc. and Algal Scientific Corporation) and paramylon derived β-1,3-glucan is also marketed as a health supplement. More specialised *E*. *gracilis* products are expected to follow [30]. The results presented by this research will facilitate process development focusing on individual products from *E*. *gracilis*. For example, we found that high concentrations of paramylon can be produced in phototrophic conditions, although previously it had been reported this was only possible in mixo- and heterotrophic conditions [4]. In addition to natural products, potential new target products from *E*. *gracilis* are being identified by transcriptomics [29], and the production of such can be realised by metabolic engineering of *E*. *gracilis* [58].

## Supporting information

S1 Fig Concentrations of individual FAMEs in photoautotrophic cultures of *E*. *gracilis* at 23 °C and 30 °C. (TIF)

S2 Fig Concentrations of individual FAMEs in heterotrophic and mixotrophic cultures of *E*. *gracilis* grown at 30 °C. (TIF)
