# Supplementary figures and images for: Euglena gracilis growth and cell composition under different temperature, light and trophic conditions
Source: PLoS One. 2018 Apr 12;13(4):e0195329. doi: 10.1371/journal.pone.0195329 (PMC5896972; doi:10.1371/journal.pone.0195329)

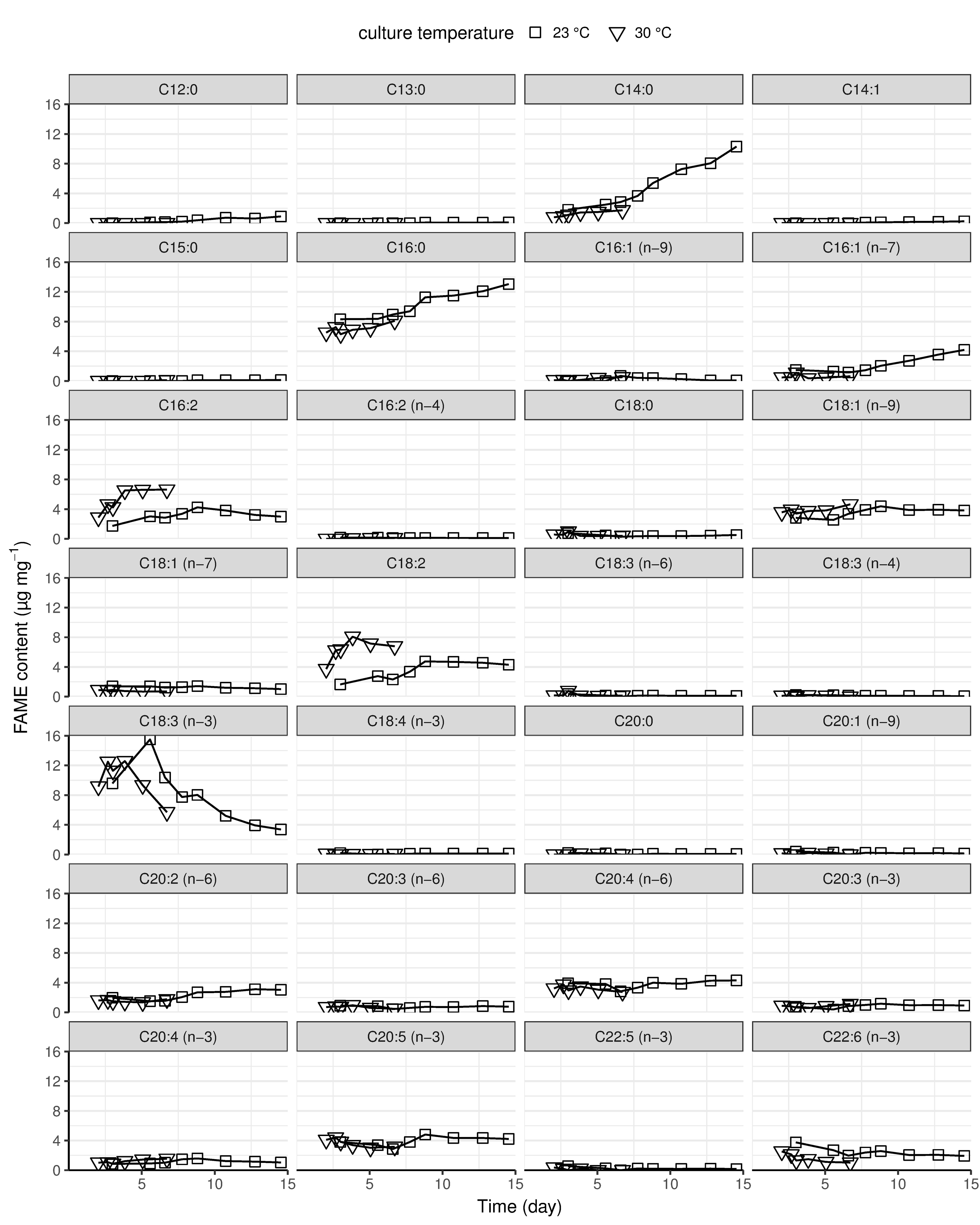

Supplement: S1 Fig — (TIF) [file pone.0195329.s001.tif]

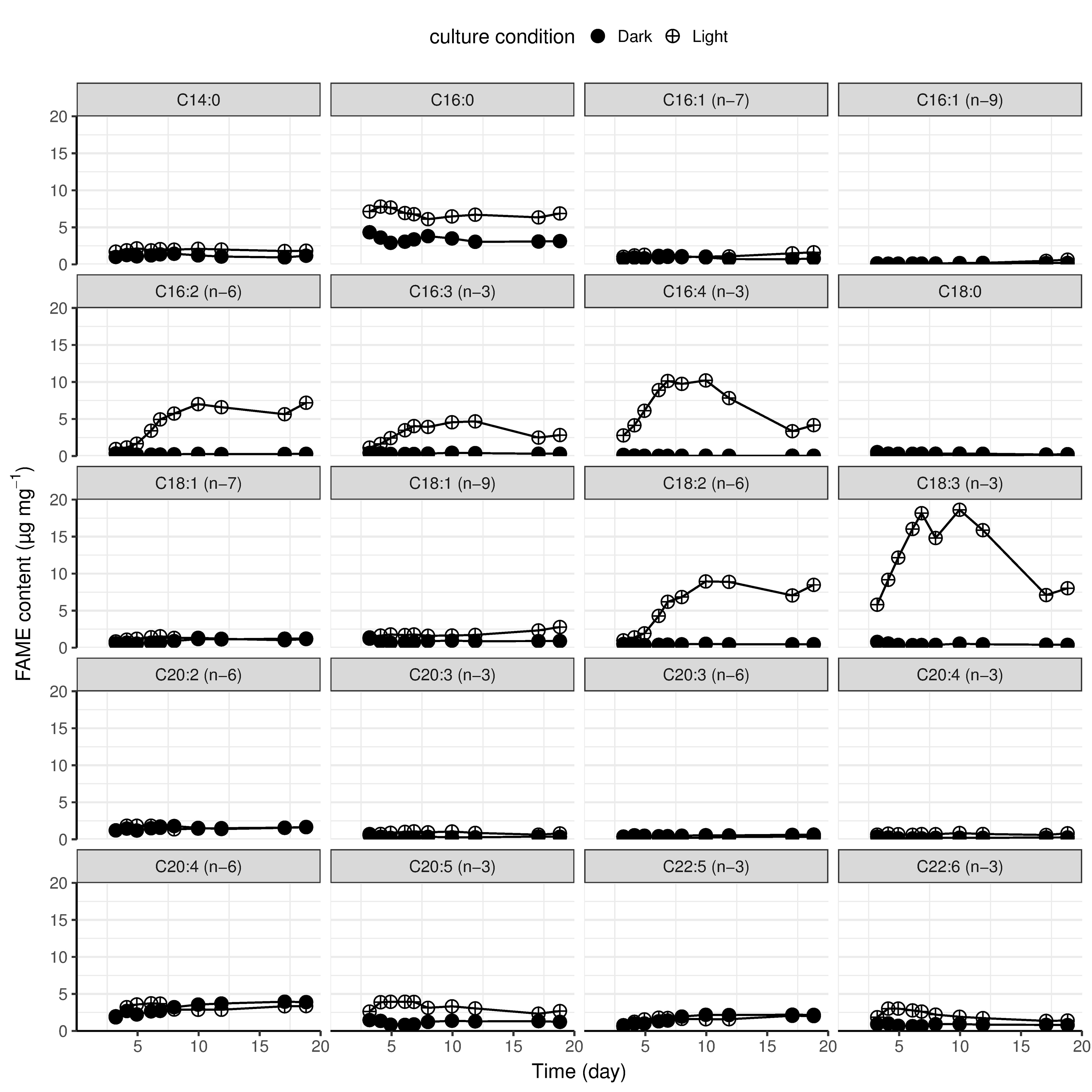

Supplement: S2 Fig — (TIF) [file pone.0195329.s002.tif]
